# Conditional relative survival among patients with chronic lymphocytic leukaemia: A population‐based study in the Netherlands

**DOI:** 10.1002/jha2.368

**Published:** 2021-12-20

**Authors:** Lina van der Straten, Mark‐David Levin, Otto Visser, Eduardus F.M. Posthuma, Jeanette K. Doorduijn, Arnon P. Kater, Avinash G. Dinmohamed

**Affiliations:** ^1^ Department of Research and Development Netherlands Comprehensive Cancer Organisation (IKNL) Utrecht The Netherlands; ^2^ Department of Internal Medicine Albert Schweitzer Hospital Dordrecht The Netherlands; ^3^ Laboratory Medical Immunology, Department of Immunology Erasmus MC Rotterdam The Netherlands; ^4^ Department of Registration Netherlands Comprehensive Cancer Organisation (IKNL) Utrecht The Netherlands; ^5^ Department of Internal Medicine Reinier The Graaf Hospital Delft The Netherlands; ^6^ Department of Hematology Leiden University Medical Center Leiden The Netherlands; ^7^ Erasmus MC Cancer Institute, Department of Hematology University Medical Center Rotterdam Rotterdam The Netherlands; ^8^ Amsterdam UMC, University of Amsterdam, Department of Hematology Cancer Center Amsterdam, Lymphoma and Myeloma Center Amsterdam Amsterdam The Netherlands; ^9^ Erasmus MC, Department of Public Health University Medical Center Rotterdam Rotterdam The Netherlands; ^10^ Amsterdam UMC, Vrije Universiteit Amsterdam, Department of Hematology Cancer Center Amsterdam Amsterdam The Netherlands

**Keywords:** cancer epidemiology, chronic lymphocytic leukaemia, conditional survival, population‐based registry, relative survival

## Abstract

Studies on conditional relative survival (CRS) in chronic lymphocytic leukaemia (CLL) have hitherto been lacking in the literature. We predicted up‐to‐date estimates of 5‐year RS at diagnosis and for each additional year survived (i.e., CRS) up to 15 years post‐diagnosis among CLL patients diagnosed during 2007–2020. We showed that 5‐year CRS continues to decline gradually with each additional year survived in a contemporary era with access to novel‐based agents, irrespective of age. This finding indicates that CLL patients continue to experience substantial excess mortality compared to an age‐ and sex‐matched group from the general population.

Chronic lymphocytic leukaemia (CLL) is the most prevalent leukaemia that predominantly affects the elderly population. It has heterogeneous natural history, ranging from an asymptomatic disease requiring no therapy for many years to an advanced disease requiring prompt therapy. The past decades have witnessed significant therapeutic advances in CLL. Population‐based studies corroborated these advances, showing a steady improvement in relative and overall survival over time across all age segments[1, 2, 3, 4, 5]. Nevertheless, these population‐based studies also highlighted that excess mortality in CLL is still an ongoing threat in modern times. This finding is not entirely surprising because most CLL patients will not be cured.

Survival estimates for patients with CLL are ordinarily measured from diagnosis or treatment. However, such estimates preclude a dynamic assessment of survival to inform long‐term CLL survivors about their current prognosis after surviving several years post‐diagnosis. This dynamic assessment of survival is often referred to as conditional survival[6]. This estimate can also be corrected for the life expectancy of the general population; that is, conditional relative survival (CRS)[6].

At present, published statistics on CRS in CLL are inherently sparse and outdated and do not report on survival estimates in elderly patients[7, 8]. We addressed this apparent knowledge gap by predicting up‐to‐date estimates of 5‐year RS at diagnosis and for each additional year survived up to 15 years post‐diagnosis among CLL patients in the Netherlands.

We selected all patients diagnosed with CLL between 1989 and 2018—with survival follow‐up through December 31, 2020—from the Netherlands Cancer Registry (NCR) that includes all newly diagnosed malignancies in the Netherlands since 1989 with a nationwide coverage of >95%[9]. The International Classification of Diseases for Oncology morphology code 9823 was used to select patients with CLL from the NCR. Details about the registry are published elsewhere[1]. Seventy‐one patients diagnosed at autopsy were excluded. The Privacy Review Board of the NCR approved the use of anonymous data for this study.

We computed 5‐year RS at diagnosis and 5‐year CRS for each additional year survived up to 15 years post‐diagnosis. RS approximates disease‐specific survival without needing information on the cause of death, which is not ascertained within the NCR. It is computed as the ratio of observed to expected survival, of which the latter is derived from Dutch population life tables and matched to patients by age, sex, and calendar year[10]. The expected survival was estimated as per the Ederer II methodology[11].

We estimated RS for patients alive during a specific period window of interest using the hybrid approach, enabling the prediction of up‐to‐date survival statistics[6]. This approach is suitable when incidence data lag behind mortality statistics, which is the case in the current study. The hybrid approach is empirically validated and conceptually similar to approaches that estimate life expectancy at birth[6]. The period window of interest was set at the follow‐up interval 2007–2020, resulting in 20 years of follow‐up post‐diagnosis for patients diagnosed during 1989–2018 who were alive at some point during 2007–2020. The survival estimates can be interpreted as the predicted survival probabilities up to 20 years post‐diagnosis for patients diagnosed during 2007–2020. Further details about the hybrid approach are delineated in the Supporting Information.

Survival estimates, with associated 95% confidence intervals (CIs) and standard errors (SEs), were calculated for the overall cohort and according to sex and age at diagnosis (i.e., 18–65, 66–75, and >75 years). Excess mortality was regarded minimal when 5‐year RS exceeded 95%[12]. Differences in survival estimates were considered statistically significant when the 95% CI did not overlap. The survival estimates were considered reliable when the SE was ≤5%. Therefore, only estimates with an SE of ≤5% were presented. Analyses were performed using STATA Statistical Software version 16.1 (StataCorp, College Station, TX, USA).

A total of 23,631 patients (61% males; median age, 69 years) were diagnosed with CLL in the Netherlands between 1989 and 2018. Table 1 summarizes the number of patients at risk for the survival analysis under the hybrid approach and the projected estimates of 5‐year RS at diagnosis and 5‐year CRS after 5, 10, and 15 years post‐diagnosis according to the baseline characteristics. Figure 1 shows the 5‐year CRS with each additional year survived up to 15 years post‐diagnosis for the overall cohort and according to baseline characteristics.

**TABLE 1:** Five‐year relative survival at diagnosis and 5, 10, and 15 years post‐diagnosis among patients with chronic lymphocytic leukaemia in the Netherlands according to baseline characteristics

	Number of patients at diagnosis		Number of patients at risk under the hybrid approach after *x* year				Conditional 5‐year relative survival (95% CI)			
Characteristics	*N*	(%)	0	5	10	15	At diagnosis	At 5 years	At 10 years	At 15 years
**Total number of patients**	23,631	–	17,235	10,562	5,193	2,120	87% (86%–88%)	84% (83%–85%)	83% (81%–85%)	81% (78%–84%)
**Sex**										
Male	14,360	(61)	10,397	6,187	2,907	1,126	86% (85%–87%)	83% (81%–84%)	82% (79%–84%)	79% (74%–83%)
Female	9,271	(39)	6,838	4,375	2,286	994	88% (87%–90%)	86% (84%–88%)	84% (82%–87%)	83% (79%–88%)
**Age at diagnosis**										
≤65 years	8,805	(37)	7,217	5,299	3,098	1,490	93% (92%–94%)	88% (87%–89%)	88% (86%–89%)	83% (80%–86%)
66–75 years	7,864	(33)	5,656	3,448	1,605	560	88% (87%–90%)	82% (80%–84%)	77% (73%–81%)	72% (64%–81%)
>75 years	6,962	(30)	4,362	1,775	490	70	75% (73%–78%)	77% (73%–82%)	—*	—*

Abbreviations: CI, confidence interval.

* Survival estimates are not reported since the standard error exceeds 5%.

**FIGURE 1:**
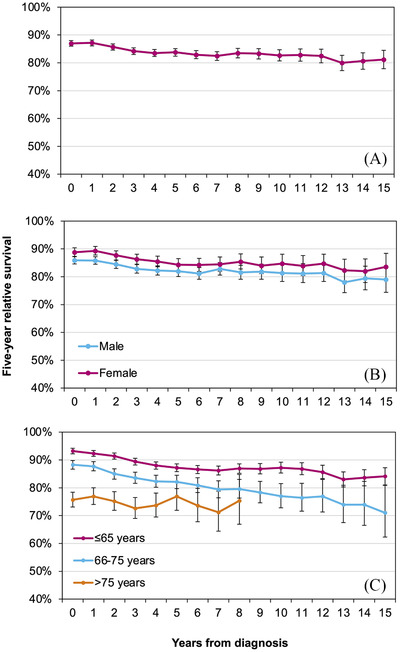
Conditional 5‐year relative survival up to 15 years post‐diagnosis among patients with chronic lymphocytic leukaemia in the Netherlands according to (A) the overall cohort, (B) sex, and (C) age at diagnosis.

Five‐year RS at diagnosis was 87% (95% CI, 86%–88%) for the overall cohort. With additional years survived post‐diagnosis, 5‐year CRS decreased slightly—ultimately reaching 81% (95% CI, 78%‐84%) at 15 years post‐diagnosis (Figure 1A). This pattern was observed regardless of sex (Figure 1B) and age (Figure 1C), with no significant sex differential in survival throughout the follow‐up period (Figure 1B). However, there was an age differential in survival that largely persisted over time (Figure 1C). Of note, 5‐year RS at diagnosis was higher for patients aged ≤65 years (93%; 95% CI, 92%–94%) and 66–75 years (88%; 95% CI, 87%–90%) compared with patients aged >75 years (75%; 95% CI, 73%–78%; Figure 1C).

At present, only two population‐based studies assessed CRS in CLL. One study included patients diagnosed in Canada between 1992 and 2006[7], whereas the other included patients diagnosed in the United States between 1973 and 2004[8]. The former study did not report age‐specific estimates of CRS, whereas the latter study restricted their analysis to patients below age 70 at diagnosis[8]. Our current analysis fills the knowledge gap by (1) predicting survival for patients diagnosed during 2007–2020 with 20 years of post‐diagnostic follow‐up information and (2) presenting survival estimates across all age segments.

Five‐year RS at diagnosis was higher in our study (85%) compared to the Canadian (77%) and United States series (62%). As for CRS, it remained stable up to 5 years post‐diagnosis in the Canadian study, whereas it slightly decreased over time in the United States study without a clear age differential in survival[7, 8]. The continuing excess mortality with each additional year survived post‐diagnosis among CLL patients may be attributed to the lack of treatment with curative potential for a broader patient population and the heightened risk of second primary malignancies, Richter's transformations, and infections and the possible occurrence of auto‐immune diseases (e.g., auto‐immune hemolysis and immune‐mediated thrombocytopenia)[13]. Also, the clinical heterogeneity of CLL could contribute to this phenomenon. More specifically, CLL can behave aggressively at diagnosis requiring immediate treatment, whereas some early‐stage CLL patients might live for many years without the need for treatment. Indeed, a study from the United States based on linked Surveillance, Epidemiology, and End Results‐Medicare data among untreated patients with CLL aged ≥66 years showed that 5‐year CRS increased modestly with each additional treatment‐free year.[14]. Of note, the previous notion of a continuous decline in CRS contrasts patterns observed in most hematological and solid malignancies that can be potentially cured, thereby highlighting the largely incurable nature of CLL[12].

Age is a well‐established prognostic factor in CLL and is used to guide treatment decision‐making. Also, it is incorporated as a dichotomized covariate (i.e., ≤65 and > 65 years) in the international prognostic index for CLL (CLL‐IPI) [15]. Our data underscores that over 75 year olds mainly drove the poor prognostic effect of age in this latter category. Therefore, age >75 years might be considered an additional variable in prognostic models, such as the CLL‐IPI, to enhance prognostication. The substantial excess mortality in elderly CLL patients that persisted during the follow‐up might be related to the reluctance to use efficacious treatment regimens due to concerns about the patients’ fitness, comorbidities, treatment‐related adverse events, or a combination of these factors. Collectively, this finding warrants further research to improve both short‐ and long‐term outcomes in, but not limited to, elderly CLL patients.

The strength of our study includes the use of a large population‐based cohort that enabled the estimation of long‐term and up‐to‐date survival among CLL patients. Also, we increased the recency of our conditional survival estimates by employing the hybrid approach. More specifically, comparable estimates derived from cohort‐based approaches would have only captured the survival experience of CLL patients diagnosed between 1989 and 2000 with 20 years of follow‐up. Therefore, the hybrid approach enables to capture recent advances in newly diagnosed patients, as well as long‐term survivors, to predict long term and up‐to‐date estimates of RS. Limitations of our study mainly pertain to the lack of detailed information on baseline clinical and molecular features. Consequently, it was not possible to stratify CRS according to well‐known prognostic indices (e.g., clinical stage as per Rai and immunoglobulin heavy chain gene and *TP53* mutational status). Also, information on the cause of death was not available to inform about the causes of excess mortality. In summary, in this nationwide, population‐based study, CRS in CLL continuously, albeit gradually, declined within each additional year survived post‐diagnosis, irrespective of sex and age. This finding indicates that excess mortality compared to the general population persists for long‐term CLL survivors. Based on this phenomenon, CLL can be generally considered an indolent disease, albeit with an insidious nature. CRS statistics provides patients with CLL and their physicians with objective information about the prognosis during follow‐up, which, in turn, can be used to guide surveillance and follow‐up activities in concert with other information on risk factors.

## CONFLICT OF INTEREST

The authors declare that they have no conflict of interest.

## AUTHOR CONTRIBUTIONS

Avinash G. Dinmohamed designed the study. Lina van der Straten analyzed the data. Avinash G. Dinmohamed supervised the data analyses. Otto Visser was responsible for the data collection. Lina van der Straten wrote the manuscript with contributions from all authors, who also interpreted the data, and read, commented on, and approved the final version of the manuscript.

## Supporting information

Supporting Information
